# The Homeodomain-Containing Transcription Factors Arx and Pax4 Control Enteroendocrine Subtype Specification in Mice

**DOI:** 10.1371/journal.pone.0036449

**Published:** 2012-05-03

**Authors:** Anthony Beucher, Elisabet Gjernes, Caitlin Collin, Monica Courtney, Aline Meunier, Patrick Collombat, Gérard Gradwohl

**Affiliations:** 1 Institut de Génétique et de Biologie Moléculaire et Cellulaire (IGBMC), Institut National de la Santé et de la Recherche Médicale (INSERM) U964, Centre National de Recherche Scientifique (CNRS) UMR 7104, Université de Strasbourg, Illkirch, France; 2 INSERM UMR 636, Diabetes Genetics Team, Nice, France; 3 Université de Nice-Sophia Antipolis, Laboratoire de Génétique du Développement Normal et Pathologique, Nice, France; University of Cordoba, Spain

## Abstract

Intestinal hormones are key regulators of digestion and energy homeostasis secreted by rare enteroendocrine cells. These cells produce over ten different hormones including GLP-1 and GIP peptides known to promote insulin secretion. To date, the molecular mechanisms controlling the specification of the various enteroendocrine subtypes from multipotent Neurog3^+^ endocrine progenitor cells, as well as their number, remain largely unknown. In contrast, in the embryonic pancreas, the opposite activities of Arx and Pax4 homeodomain transcription factors promote islet progenitor cells towards the different endocrine cell fates. In this study, we thus investigated the role of Arx and Pax4 in enteroendocrine subtype specification. The small intestine and colon of *Arx*- and *Pax4*-deficient mice were analyzed using histological, molecular, and lineage tracing approaches. We show that Arx is expressed in endocrine progenitors (Neurog3^+^) and in early differentiating (ChromograninA^−^) GLP-1-, GIP-, CCK-, Sct- Gastrin- and Ghrelin-producing cells. We noted a dramatic reduction or a complete loss of all these enteroendocrine cell types in *Arx* mutants. Serotonin- and Somatostatin-secreting cells do not express Arx and, accordingly, the differentiation of Serotonin cells was not affected in *Arx* mutants. However, the number of Somatostatin-expressing D-cells is increased as *Arx*-deficient progenitor cells are redirected to the D-cell lineage. In *Pax4*-deficient mice, the differentiation of Serotonin and Somatostatin cells is impaired, as well as of GIP and Gastrin cells. In contrast, the number of GLP-1 producing L-cells is increased concomitantly with an upregulation of *Arx*. Thus, while Arx and Pax4 are necessary for the development of L- and D-cells respectively, they conversely restrict D- and L-cells fates suggesting antagonistic functions in D/L cell allocation. In conclusion, these finding demonstrate that, downstream of Neurog3, the specification of a subset of enteroendocrine subtypes relies on both Arx and Pax4, while others depend only on Arx or Pax4.

## Introduction

Enteroendocrine cells belong to one of the four main intestinal cell subtypes, including enterocytes, goblet and Paneth cells, and represent about 1% of all epithelial cells. These cells secrete various amine and peptide hormones and are classified according to their main secretory product, including Glugagon-like peptide 1 (GLP-1), Glugagon-like peptide 2 (GLP-2) and Peptide YY (PYY) secreted by L-cells, Gastric inhibitory peptide (K-cells), Somatostatin (D-cells), Cholecystokinin (I-cells), Secretin (S-cells), Gastrin (G-cells), Serotonin (EC cells), and Neurotensin (N-cells). The gastric peptide Ghrelin is also found in the small intestine and colon [1], [2], but it remains unclear whether intestinal Ghrelin-expressing cells constitute a separate enteroendocrine subtype. Intestinal hormones control numerous physiological functions, such as glucose homeostasis for the Glucoincretin GLP-1 and GIP, food intake, pancreatic and gastric secretion, or gastrointestinal mobility [3], [4]. In mice, the loss of all enteroendocrine cells leads to growth retardation, impaired lipid absorption and increased lethality, underlying the importance of enteroendocrine function [5].

During the course of development, enteroendocrine cells, as well as the two other secretory cell types, goblet and Paneth cells, arise from intestinal stem cells through an intermediate progenitor expressing the basic helix-loop-helix (bHLH) transcription factor (TF) Atoh1 [6], [7]. The specification of this Atoh1^+^ secretory progenitor cell towards the endocrine lineage is controlled by the bHLH TF Neurog3 that also determines endocrine cell destiny in the stomach and pancreas [8], [9], [10]. Downstream of Neurog3, several TFs have been shown to be required for proper enteroendocrine cell differentiation. Among these, the zinc-finger TF Insm1 is necessary for generic features of endocrine cells as well as for the differentiation of particular subtypes. Indeed, targeted disruption of Insm1 leads to the loss of expression of Chromogranin A (ChgA) secretory vesicle protein [11]. Hormone expression is only partially affected by the absence of Insm1. Substance P and Neurotensin (Nts) cells are lost but the numbers of Serotonin (5HT), Cholecystokinin (CCK) and PYY cells are only reduced. Additional TFs were found to control the allocation towards specific enteroendocrine subtypes. Thus, NeuroD1 controls the differentiation of Secretin (Sct) and CCK cells [12], whereas Foxa1 and Foxa2 promote the differentiation of GLP1- and Somatostatin- (Sst-) expressing cells [13]. The NK-homeodomain-encoding gene, Nkx2.2, is necessary for CCK, Gastrin, Gastric Inhibitory Polypeptide (GIP), Nts and Sst expression [14]. The paired-box transcription factors Pax4 and Pax6 also control enteroendocrine cell differentiation. Although Pax6 knock out phenotype has not been extensively investigated in the intestine, it has been reported that GIP cells are Pax6-dependant [15]. Other studies indicate that Pax6 acts downstream and in concert with Foxa1 and Foxa2 to regulate the transcription of the preproglucagon gene [13]. Pax4 inactivation results in the loss of several enteroendocrine subtypes, such as Serotonin-, Sct-, GIP-, PYY- and CCK-expressing cells [15]. Taken together, these data indicate that enteroendocrine subtype specification and differentiation rely on a complex network of transcription factors.

In the embryonic pancreas, the specification of the four main endocrine subtypes including alpha-, beta-, delta- and PP-cells is essentially under the control of the opposing actions of Arx and Pax4 TFs acting downstream of the proendocrine gene Neurog3. Mice deficient for Pax4 lack beta- and delta-cells and display a concomitant increase in alpha-cell number [16]. Conversely, Arx inactivation leads to an opposite phenotype, characterized by an absence of alpha-cells and an increase in the number of beta- and delta-cells [17]. Furthermore, the forced expression of *Arx* in endocrine progenitors induces their specification towards the alpha-/PP-cell lineages at the expense of the beta-/delta-cell fates [18]. Interestingly, the ectopic expression of Pax4 in alpha-cells is sufficient to convert these cells into beta-like cells [19]. Therefore, the decision between the alpha-/PP- or beta-/delta-cell fate seems to be mainly directed by the cross-repression of *Pax4* and *Arx* genes [20]. Thus, the balance between Arx and Pax4 in pancreatic endocrine progenitors plays a key role in endocrine subtype allocation.

Since Arx and Pax4 control islet subtype destiny in the developing pancreas, we postulated that similar mechanisms could govern cell fate choices in the enteroendocrine lineage. In this study, we therefore investigated the function of Arx and Pax4 in the intestine. Our results indicate that Arx is restricted to the enteroendocrine lineage and downstream of Neurog3. Importantly, Arx is required for the differentiation of a subset of enteroendocrine cells. Indeed, *Arx*-deficient mice display an almost complete loss of GLP1, GIP, CCK and Nts cells, with a concomitant increase in Sst-expressing cell numbers. On the other hand, *Pax4*-deficient mice lack Sst, GIP and Serotonin cells, whereas GLP1 cell number is significantly increased. Taken together, these results indicate that while Arx and Pax4 are similarly required for the proper differentiation of a subset of enteroendocrine cells, they differentially regulate the development of specific enteroendocrine cells. In contrast to the embryonic pancreas antagonistic functions of Arx and Pax4 seems limited to the control of L (GLP-1)- and D (Sst)-cell differentiation.

## Results

### Arx is transiently expressed in a subset of developing enteroendocrine cells

**Figure 1:**
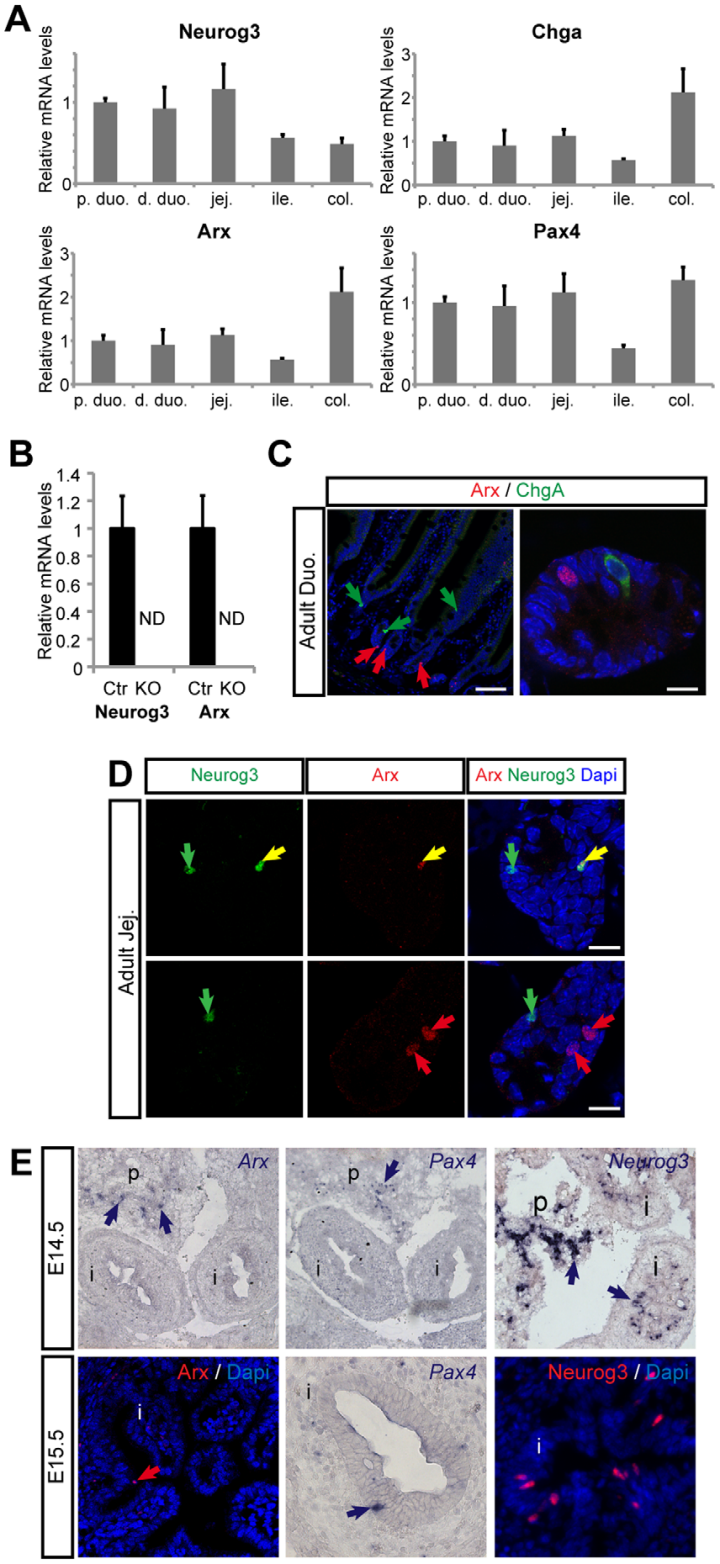
Arx is expressed in enteroendocrine precursors, downstream of Neurog3. (A) Real time RT-PCR analysis of *Neurog3*, *ChgA*, *Arx* and *Pax4* expression in different intestinal regions of 8 weeks old wild-type mice (n = 3). (B) Real time RT-PCR analyses of *Neurog3* and *Arx* expression in 8–10 weeks old Villin-Cre;Neurog3^f/f^ (KO) mice and control Villin-Cre;Neurog3^f/+^ (Ctr) mice. *Arx* expression is completely lost in absence of Neurog3 (n = 5). (C–D) Immunofluorescence on sections of wild-type adult duodenum (C,) and jejunum (D). In C, Arx^+^ cells (red arrows) are localized in the crypt and are ChgA-negative (ChgA^+^ cells, green arrows). In D, Partial overlapping expression of Arx and Neurog3 in the adult mouse intestine is illustrated. Yellow, green and red arrows point to double-labeled, single Neurog3^+^ and single Arx^+^ cells, respectively. (E) *In situ* hybrization and Immunofluorescence on cross sections of wild-type embryonic pancreas (p) and intestine (i). Blue arrows point to cells expressing *Arx*, *Pax4* or *Neurog3* transcripts. Arx and Pax4 expressions are detected 24 h after Neurog3 expression in enteroendocrine precursors. The red arrow points to an Arx expressing cell. p., proximal; d., distal; duo., duodenum; jej., jejunum; ile., ileum; col., colon; SI, small intestine; p, pancreas; I, intestine. Values are means ± SD. Scale bars (C, left panel) 50 µm, (C right panel, D) 10 µm. ND, Not Detected.

**Figure 2:**
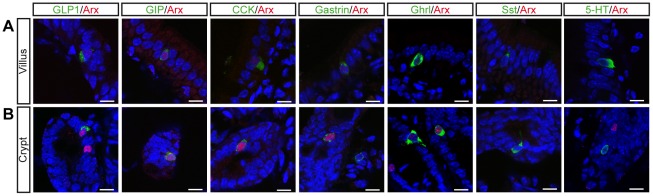
Arx is expressed in early differentiating GLP1-, GIP-, CCK- and Gastrin-expressing cells in the adult small intestine. Co-immunostaining with Arx and intestinal hormones antibodies on sections of adult small intestine. Arx is strongly expressed in GLP1^+^, GIP^+^, CCK^+^, and selected Ghrl^+^ cells located in the crypts (B), but not in Sst^+^ or Serotonin^+^ (5-HT) cells. Arx expression level decreases in enteroendocrine cells in the villi (A). Scale bar 10 µm.

To characterize the expression pattern of Arx in the embryonic and adult mouse intestine, we combined quantitative RT-PCR, *in situ* hybridization and double immunohistochemistry using antibodies raised against Arx, Neurog3, ChromograninA, and intestinal peptides. In the adult wild-type intestine, *Arx* transcripts are revealed from the duodenum to the colon (Fig. 1A). Importantly, *Arx* transcripts cannot be detected in the duodenum of Villin-Cre; Neurog3^f/f^ mice (Fig. 1B), which lack enteroendocrine cells [5]. This suggests that, like in the pancreas [17], *Arx* expression remains restricted to the endocrine lineage in the intestine. Accordingly, scattered Arx^+^ cells are found throughout the adult intestine in a pattern reminiscent of enteroendocrine cells (Fig. 1C, S1). In the small intestine, Arx is expressed in post-mitotic crypt cells (Fig. S2), mainly in subsets of Neurog3^+^ cells (Fig. 1D), suggesting that Arx expression is initiated in endocrine progenitor cells. Arx is not detected in mature ChgA^+^ endocrine cells (Fig. 1C), however cells double-positive for Arx and intestinal peptides GLP1, GIP, CCK, Gastrin or Ghrelin (Ghrl) are present within the crypts, supporting the notion that Arx expression is maintained in early differentiating L-, K-, I-, G- and Ghrelin-cells (Fig. 2). As Arx-positive cells migrate during their differentiation to reach the base of the villus, Arx expression progressively diminishes and eventually vanishes (Fig. 2 compare A to B), further suggesting that Arx is expressed in nascent but not mature hormone-expressing cells. Importantly, Arx is never detected in Somatostatin- nor Serotonin-expressing D or EC cells respectively (Fig. 2). During embryogenesis, at E14.5 when endocrine commitment is initiated in Neurog3+ cells, *Arx* expression is not detectable. However, around E15.5, Arx-expressing cells emerge in the embryonic intestine, at a stage corresponding to the onset of endocrine differentiation (Fig. 1E). *Arx* transcripts are not detected in Neurog3-deficient embryonic intestines (data not shown) and thus, like in the adult, Arx expression is restricted to the enteroendocrine lineage. Taken together, these data indicate that in the embryonic intestine Arx lies downstream of Neurog3 in endocrine committed cells. In the adult intestine Arx appears transiently expressed downstream of Neurog3 in endocrine progenitors and developing, but not fully differentiated, L-, K-, I-, G- and Ghrelin-cells, whereas D- and EC-cells do not appear to arise from Arx^+^ precursors.

### Enteroendocrine cell differentiation is severely impaired in *Arx*-deficient mice

**Figure 3:**
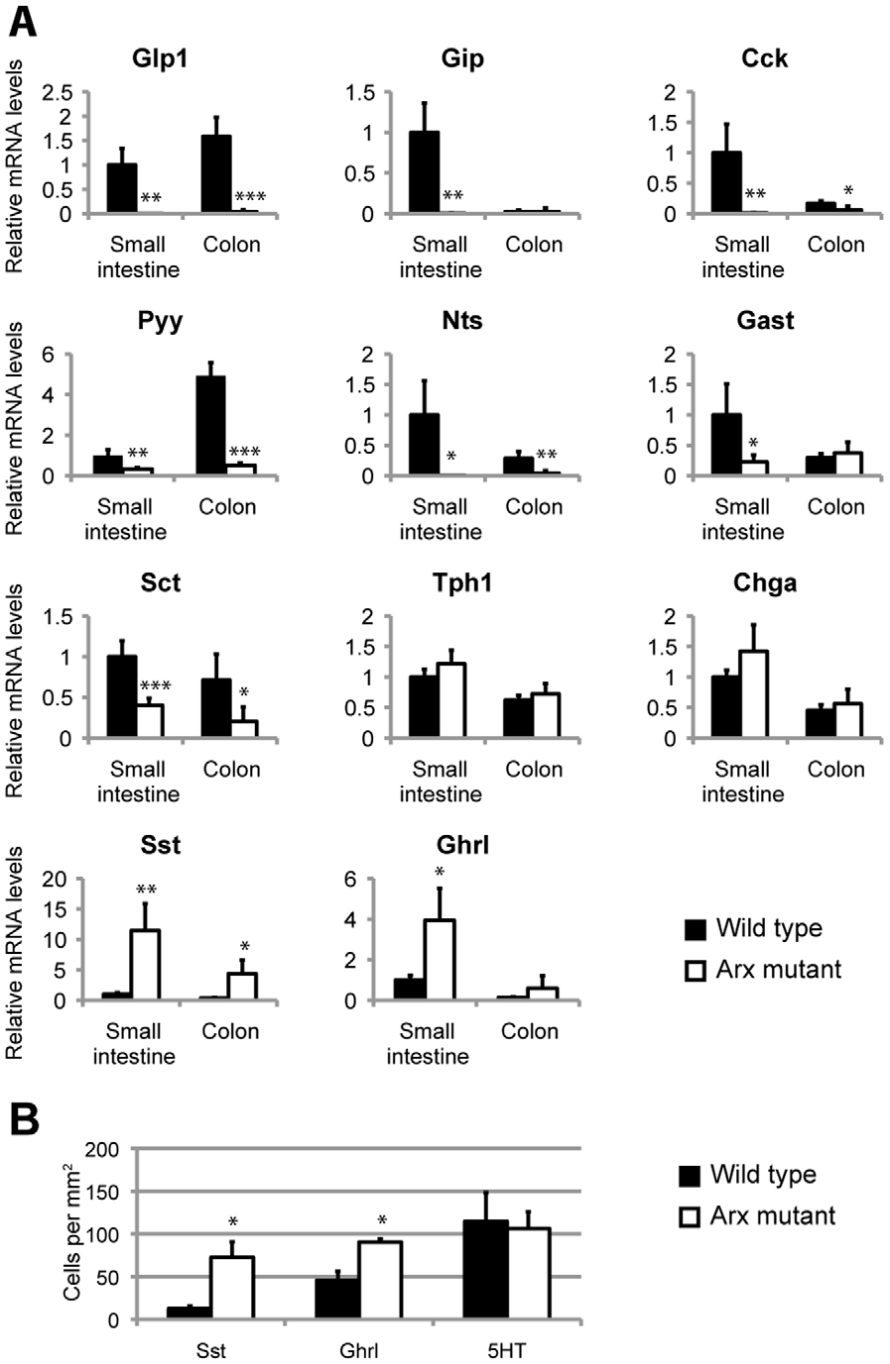
Hormone expression in *Arx*-deficient intestine. (A) Real time RT-PCR analyses of various intestinal hormones mRNAs in *Arx*-deficient and control small intestine and colon at 2 days *postpartum* (n = 5). *Glp1*, *Gip*, *Cck*, *Pyy*, *Nts* and *Sct* mRNA levels are significantly reduced in Arx mutant mice, whereas *Sst* and *Ghrl* expression are increased in the small intestine. (B) Quantification of Sst^+^ and Ghrl^+^ cells in Arx^+/+^ (n = 3) and Arx^−^ P1 duodenum (n = 3). Both Sst and Ghrl-expressing cell numbers increase in *Arx*-deficient duodenum while the number of Serotonin-cells (5HT) is unchanged. Student's T-test *p<0.05, **p<0.01, ***p<0.001.

**Figure 4:**
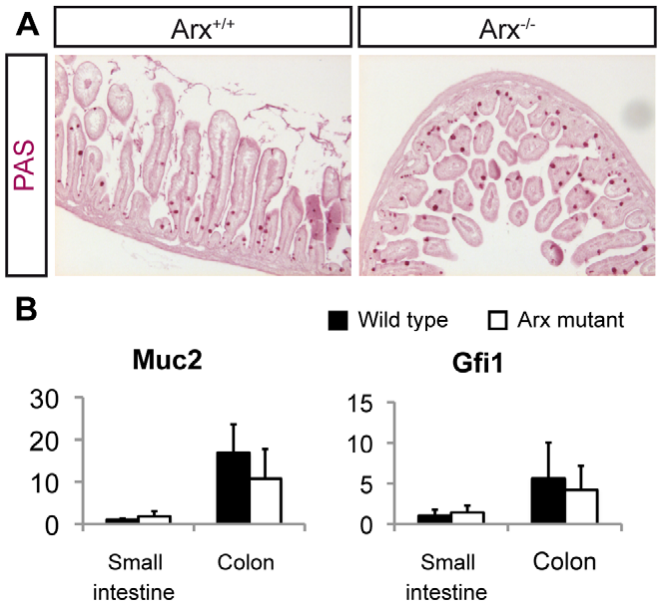
Normal goblet cell differentiation in *Arx*-deficient mice. (A) Periodic Acid Schiff (PAS) staining showing PAS^+^ goblet cells in wild type and *Arx*-deficient newborn intestine. (B) mRNA quantification of the goblet cell marker *Muc2* and *Gfi1*, a key TF regulating goblet cell specification, in Arx mutant intestine at P2. The expression of *Muc2* and *Gfi1* is not statistically different between *Arx*-deficient intestines (n = 5) and controls (n = 5).

We next analyzed enteroendocrine cell differentiation in *Arx*-deficient mice. Arx mutants do not survive beyond postnatal day 2 (P2). We therefore examined intestinal hormone expression combining real-time PCR (Fig. 3A and Table S1) with immunofluorescence (Fig. 3B, S3) at P1-P2. *Arx-deficient* mice display an almost complete absence of *Glp1-*, *Gip-*, *Cck-* and *Nts*-expressing cells in the small intestine and colon (Fig. 3 and S3A), whereas *Sct* and *Gastrin* mRNA levels are significantly diminished. *Pyy*, which is normally expressed in GLP1 cells (L cells), is also found drastically reduced. In contrast, we observed a significant increase of Sst^+^ and Ghrl^+^ cell numbers (5.64±1.41 and 1.97±0.08 fold increase in the duodenum, respectively; Fig. 3A,B), while *Tph1* expression, a marker of Serotonin-expressing EC cells, is unchanged. In agreement with the RT-QPCR data, the numbers of Serotonin-expressing cells are similar in controls and mutants (Fig. 3B). Interestingly, *ChgA* expression is also unaffected in the Arx mutant intestine, suggesting that the overall number of enteroendocrine cells is not altered. As suggested by the restriction of Arx expression to the endocrine lineage, Arx inactivation does not alter the differentiation of Goblet cells (Fig. 4 and data not shown). Thus, our results demonstrate that *Arx* is necessary for the differentiation of GLP-1-, GIP-, CCK-, Sct- Gastrin- and Nts-expressing cell lineages and suggest that failed cells, would to some extend, develop into Sst^+^ and Ghrl^+^ cells.

### *Arx*-deficient progenitors are reallocated to Somatostatin-expressing cells

**Figure 5:**
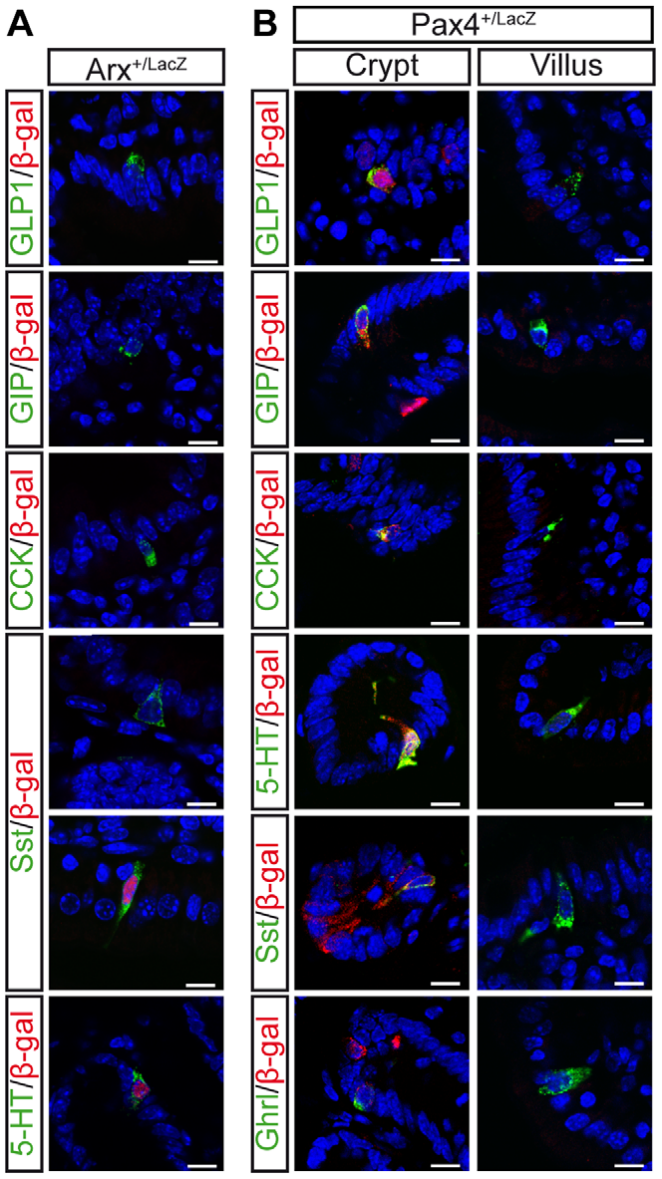
Short-term lineage tracing of *Arx*-deficient cells and *Pax4*-expressing cells. Co-immunodetection of beta-gal and intestinal hormones in the adult duodenum of Arx heterozygous females (A) and of Pax4 heterozygous mice (B). (A) The beta-gal protein was never detected in GLP1-, GIP- or CCK-cells. *Arx*-deficient cells, which express the beta-gal instead of Arx, can differentiate into Sst- or Serotonin- (5HT-) expressing cells. In Pax4 heterozygous mice (B), the beta-gal is expressed in the crypts and can be detected in all endocrine cell types. beta-gal is not expressed in endocrine cells located in the villi.

To determine whether the changes in endocrine differentiation observed upon Arx deficiency were caused by alternative fate specification, we analyzed the expression of intestinal hormones in *Arx*-deficient cells. In mice carrying an *Arx* mutant allele, the beta-galactosidase (beta-gal) protein is expressed under the control of *Arx* regulatory elements. Due to the location of *Arx* on the X chromosome, random X inactivation leads to the silencing of either the wild-type or *LacZ* Arx allele in heterozygous females (*X^ArxWt^X^ArxLacZ^*). Consequently, in *Arx^+/LacZ^* females, cells expressing the Arx protein do not express the beta-gal, and cells expressing the beta-gal are *Arx*-deficient. In the adult intestine of *Arx^+/LacZ^* female mice, we did not detect any beta-gal expression in GLP1-, GIP- or CCK-producing cells (Fig. 5A), confirming that Arx expression is required for the generation of L-, K- and I-cells. In contrast, beta-gal was found in a subset of Sst-producing cells (Fig. 5A), which do not express Arx in wild-type intestine (Fig. 2). Together with the observed increase of *Sst* mRNA (Fig. 3A) and the augmentation of Sst-expressing cell numbers (Fig. 3B), our results support the hypothesis that *Arx*-deficient progenitors, which fail to generate a large subset of enteroendocrine cells, adopt an alternative Sst-expressing cell fate. As seen for Sst^+^ cells, *Ghrelin* mRNA (Fig. 3A) and the number of Ghrelin-producing cells (Fig. 3B) increase in *Arx*-deficient intestine. However, as a subset of Ghrelin^+^ cells expresses Arx in wild-type intestine and as Ghrelin is also co-expressed with GLP1 or Sst (Fig. S4), it is impossible to ascertain the cellular mechanisms leading to the increased number of Ghrelin^+^ cells in *Arx*-deficient intestine. Interestingly, our tracing data also reveal that a subset of Serotonin-expressing cells (Fig. 5A) also derive from *Arx*-deficient cells. Based on the absence of Arx/Serotonin co-expression, we postulated that Serotonin^+^ cells do not normally arise from Arx progenitors. Thus taken together, our findings suggest that *Arx*-deficient progenitor cells could be reallocated to the Serotonin-producing EC lineage. Given the high number of Serotonin-expressing EC-cells, we hypothesize that the reallocation of some *Arx*-deficient cells towards the EC lineage is however not sufficient to significantly impact the number of Serotonin-expressing cells (Fig. 3B). In summary, we conclude that the increase in Sst-expressing cell numbers observed in Arx mutants results from the reallocation of progenitor cells to the Sst lineage rather than from the expansion of Arx-independent Sst^+^ cell precursors.

### Opposing functions of Pax4 and Arx control the specification of GLP1- (L-cells) and Somatostatin- (D-cells) expressing cells

**Figure 6:**
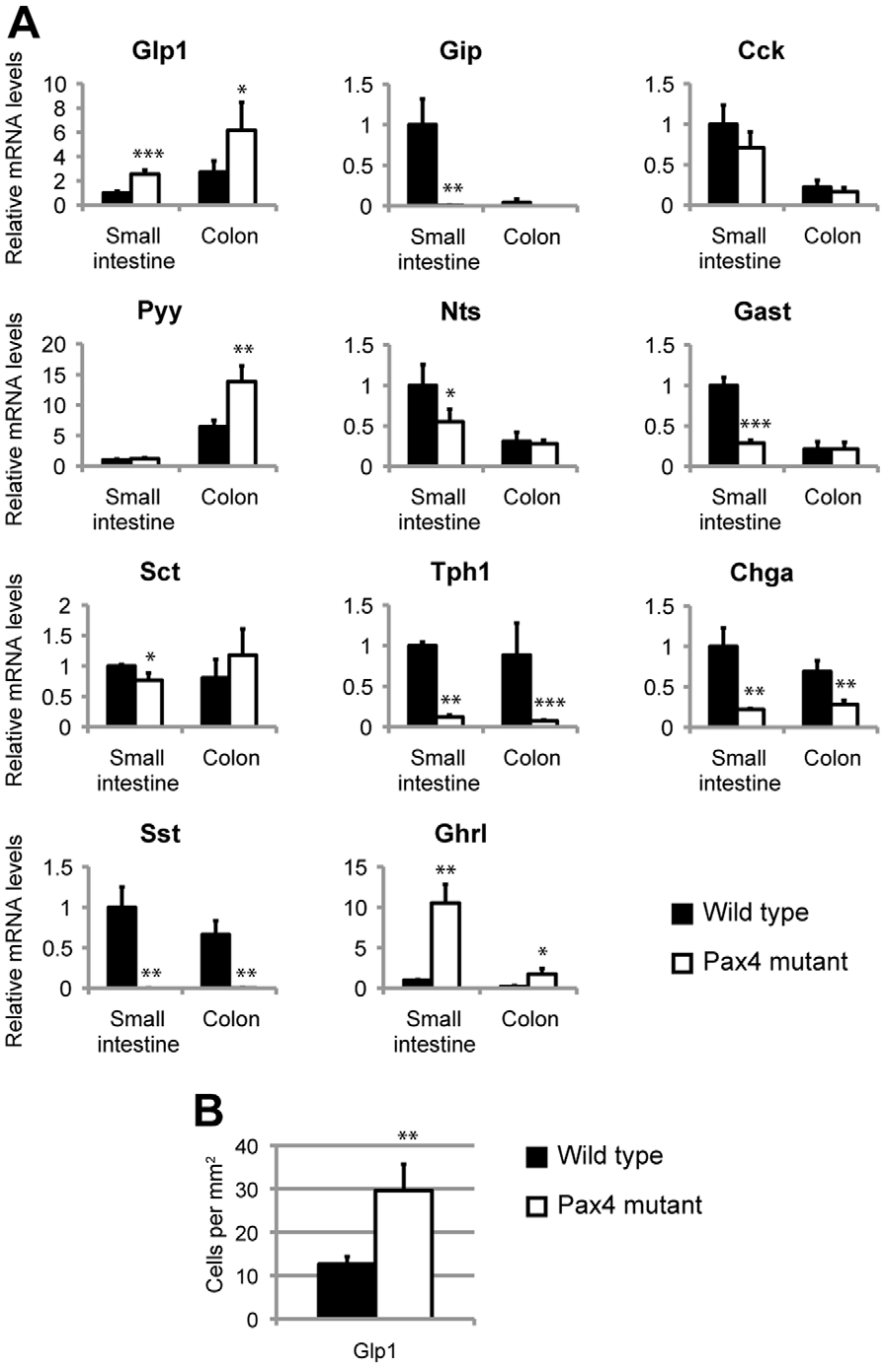
Hormone expression in *Pax4*-deficient intestine. (A) Real time RT-PCR analyses of various intestinal hormones mRNAs in *Pax4*-deficient and control small intestine and colon at 2 days *postpartum* (n = 4). *Gip*, *Nts*, *Gast*, *Sct* and *Tph1* mRNA levels decrease significantly in Pax4 mutant small intestine, *Glp1* and *Ghrl* expressions increase in both the small intestine and colon. (B) Quantification of GLP1^+^ cells in Pax4^+/+^ (n = 3) and Pax4^−/−^ P1 ileum (n = 3). GLP1-expressing cells are more abundant in Pax4 mutant ileum. Student's T-test *p<0.05, **p<0.01, ***p<0.001.

The consequences of Pax4 loss-of-function on enteroendocrine cell differentiation have previously been reported [15]. However, since the expression of several hormones, including Glp1 and Sst, as well as of downstream transcription factors was not addressed, we decided to reinvestigate the phenotype of Pax4 mutants. Firstly, we determined which endocrine subtypes express Pax4. Due to the lack of working anti-Pax4 antibodies, we took advantage of *Pax4^+/−^* (*Pax4^+/LacZ^*) mice, in which the *beta-galactosidase* gene is inserted within the *Pax4* locus [16], to label *Pax4*-expressing cells (beta-gal^+^). In adult mice, the beta-gal was co-detected with all hormones tested suggesting that *Pax4* is expressed in all enteroendocrine subtypes analyzed, including GLP1-, GIP-, CCK-, Serotonin- and Ghrelin-expressing cells (Fig. 5B). Because of the stability of the beta-gal [8], we could not determine whether Pax4 is expressed in progenitors and/or in their differentiated descendants. However, the absence of beta-gal expression in the villi suggests that *Pax4* expression is not maintained in mature endocrine cells (Fig. 5B). Analyses of Pax4 mutant mice revealed that, in contrast to *Arx*-deficient animals, *ChgA* expression in P2 small intestine and colon is severely reduced (22.1%±1.1 and 41.0%±7.1, respectively, as compared to controls; Fig. 6A). Furthermore, the differentiation of many enteroendocrine subtypes is impaired, as demonstrated by the decrease of *Nts*, *Gastrin* and *Sct* expression in the small intestine and the almost absence of *Gip* and *Tph1* expression (Fig. 6A and Table S1). Interestingly, *Sst* expression, which is increased in Arx mutants, is lost in *Pax4*-deficient mice. Conversely, *Glp1* expression is augmented in Pax4 mutants, whereas it is lost in Arx mutants (compare Fig. 3A to Fig. 6A). In addition, the concomitant increase of *Glp1* and *Pyy* transcripts in *Pax4*-deficient mice suggests an augmentation of the number of L cells. In agreement with this hypothesis, counting of GLP-1-positive cells in the ileum (P2) revealed a doubling of the number of L-cells (Fig. 6B). Finally, as previously described [21], we confirmed an increase in *Ghrl* expression in Pax4 mutants. In summary, these results indicate that Pax4, like Arx, is necessary for the proper development of GIP, Gastrin and Neurotensin cells in the small intestine while Serotonin-producing cells are exclusively Pax4-dependent. Furthermore the allocation of GLP1^+^ and Sst^+^ lineages appears to be regulated by the opposing roles of Arx and Pax4 respectively.

### *Arx* expression is upregulated in *Pax4*-deficient intestine but *Pax4* is unaltered in Arx mutant

**Figure 7:**
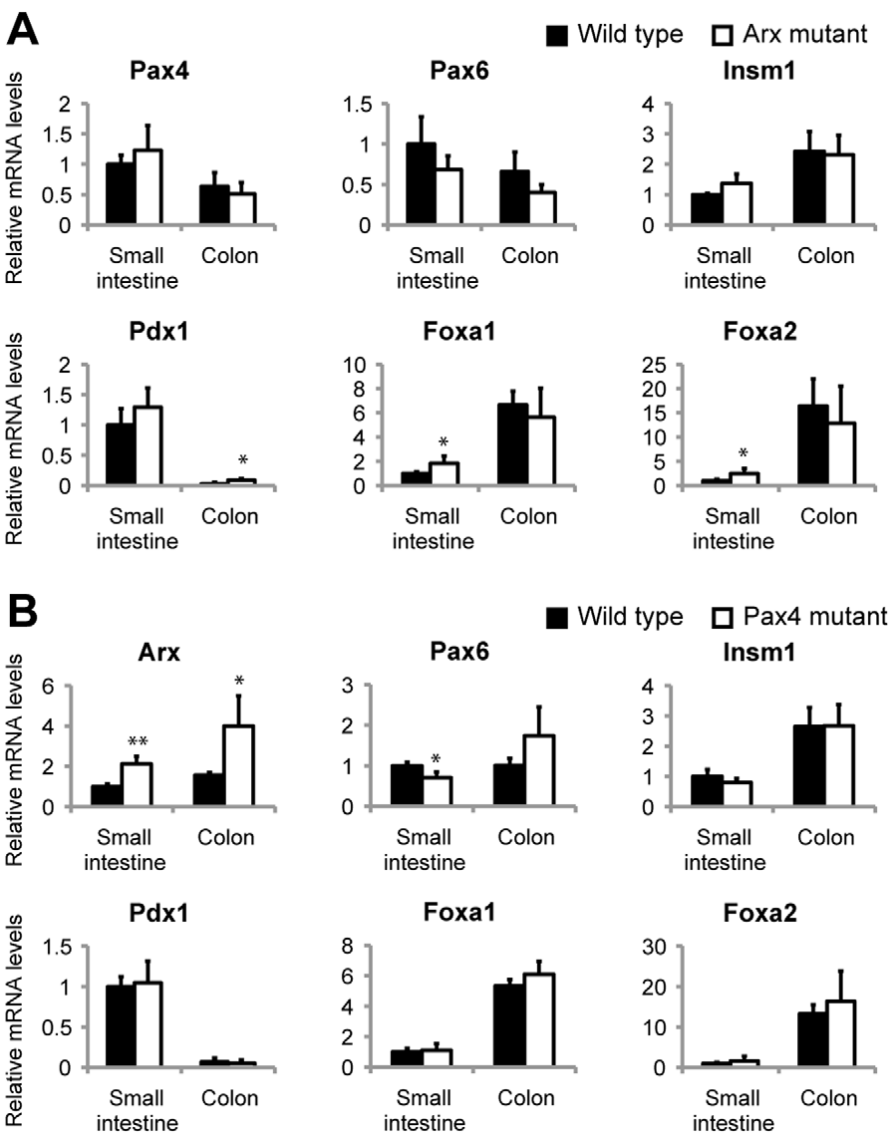
Expression of transcription factors in *Arx-* and *Pax4*-deficient intestines. Real time PCR analyses in (A) *Arx-* and (B) *Pax4-* (n = 4) deficient (n = 5) and control (n = 5) small intestine and colon at 2 days *postpartum*. (A) *Pdx1* and *Foxa1*/*a2* expression are increased in Arx mutant colon and small intestine, respectively. (B) *Arx* is significantly upregulated in Pax4 mutants. Student's T-test *p<0.05, **p<0.01, ***p<0.001.

To determine the position of Arx and Pax4 in the cascade of transcription factors implementing the enteroendocrine differentiation program, we analyzed the expression of a series of TFs in Arx- and *Pax4*-deficient intestines. The levels of *Neurog3*, *NeuroD1*, *Insm1*, *Rfx6*, *Mafb* and *Nkx2*.2 transcripts remained unchanged in both knockouts (Fig. 7 and Fig. S5). In contrast, *Foxa1* and *Foxa2* expressions were moderately but significantly increased in *Arx*-deficient small intestine as well as *Pdx1* in the colon (Fig. 7A). As the inactivation of these TFs leads to decreased *Sst* expression [13], [22], their up-regulation in Arx mutant mice could in turn promote *Sst* transcription. The expression of *Pax6*, which is generally considered to be a late TF in islet cell development, slightly decreased in Pax4 mutants but is unaffected in Arx mutants (Fig. 7). Importantly, we observed 2.1±0.37 times more *Arx* mRNA in *Pax4*-deficient small intestines compared to controls (Fig. 7B). However, we could not detect a significant increase in *Pax4* expression in Arx mutants (Fig. 7A). To determine whether either Arx or Pax4 was sufficient to induce phenotypic changes in enteroendocrine cells we performed gain of function experiments in STC-1 cell line [23]. In this experimental system, overexpression of Pax4 or Arx did not alter *Arx* or *Pax4* transcription respectively or hormone gene expression (Fig. S6). In summary the expression of many TFs is unaffected in Arx- or *Pax4*-deficient mice, suggesting that Arx and Pax4 act downstream or in parallel pathways. The strong induction of *Arx*, both in the small intestine and colon of Pax4 KO mice, suggests that Pax4 controls the specification of endocrine subtypes through the repression of *Arx*.

## Discussion

**Figure 8:**
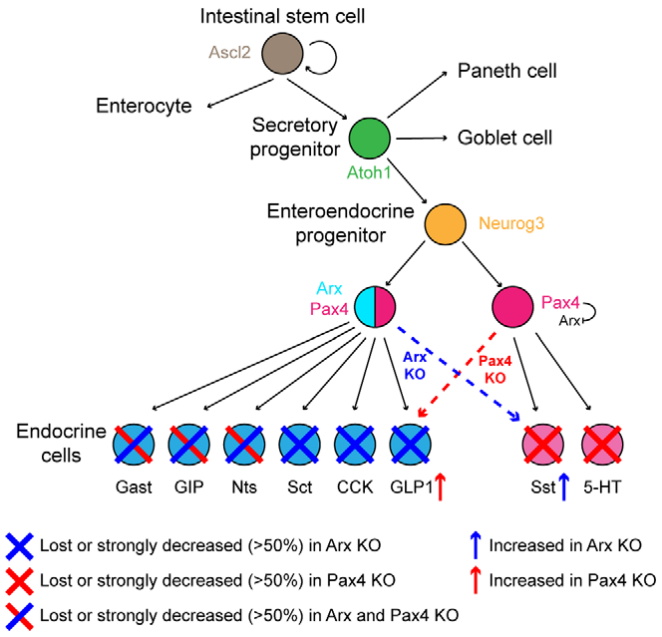
Model of enteroendocrine subtype specification during small intestine development: roles of Arx and Pax4. Gast-, GIP-, Nts-, Sct-, CCK- and GLP1-expressing cells arise from endocrine progenitors expressing Neurog3 then Pax4 and Arx. Upon Arx inactivation these progenitors are reallocated into Sst-expressing cells while the differentiation of Gast-, GIP-, Nts-, Sct-, CCK- and GLP1-expressing-cells is impaired. Sst- and Serotonin (5-HT)-expressing cells are generated from progenitors expressing Neurog3 then Pax4. Inactivation of Pax4 leads to the up-regulation of Arx and the differentiation of these progenitors into GLP1-expressing cells, while the differentiation of Sst-, Serotonin (5-HT)- Gast-, GIP- and Nts-expressing cells is impaired. Key transcription factors controlling intestinal cell destiny are also indicated.

Previous studies have demonstrated the essential role of Arx in cell fate decision during pancreas development and forebrain morphogenesis [17], [20], [24]. In this study, we showed that Arx and Pax4 are required for the differentiation of several enteroendocrine cells in the small and large intestine and control the specification of endocrine subtypes (Fig. 8). Arx expression is strictly dependent on Neurog3 demonstrating that Arx is exclusively found in the intestinal endocrine lineage and not in other intestinal cell types. We revealed Arx expression in subsets of post-mitotic Neurog3-positive endocrine progenitor cells in the embryonic and adult intestine. Arx is subsequently maintained in nascent hormone-expressing cells still located in the crypt. These include the Gluco-incretin GLP1- and GIP-expressing cells, which derive from Arx-positive progenitors, but exclude Somatostatin- and Serotonin-expressing cells. Lineage tracing experiments in wild-type mice using BAC transgenics would be required to further ascertain that Somatostatin- and Serotonin-expressing cells do not arise from Arx-expressing progenitors. Mature, Chromogranin A-positive, endocrine cells present in the villi are devoid of Arx. In summary, our results suggest that *Arx* transcription is switched on in selected endocrine progenitors to control their destiny. *Arx* is then transiently expressed in early hormone-expressing cells and subsequently switched off. This expression pattern contrasts with the observation made in the pancreas where the Arx protein remains expressed in mature alpha-cells in adult islets [19] and, importantly, suggests that Arx controls the differentiation of enteroendocrine cells but not their function.

While we observed a loss or drastic reduction of several enteroendocrine cell populations in *Arx*-deficient intestines, Secretin- or Gastrin-expressing cells were only found to be reduced. This suggests that an Arx-independent program can lead to the differentiation of Secretin- or Gastrin-cells. Interestingly, we observed a very significant increase of both *Sst* mRNA and somatostatin-expressing cell numbers. On the other hand, the expression of *ChgA*, encoding a component of the secretory granules of most enteroendocrine cells, was found unchanged, suggesting that the total enteroendocrine cell number is unaffected. Taken together, these data indicate that, upon Arx deficiency, a reallocation of developing enteroendocrine progenitor cells towards the somatostatin lineage occurs.

The identification of Somatostatin^+^/Arx^−^ cells in tracing experiments further supports the notion of a cell type conversion rather than a function of Arx in the repression of the transcription of the *Sst* gene. Notably, the numbers of Somatostatin-expressing delta-cells (in addition to beta-cells) are also increased in *Arx*-deficient pancreata, suggesting a similar function of Arx in the repression of the differentiation program leading to the generation of somatostatin-producing cells. It is tempting to speculate that a similar reallocation occurs towards Ghrelin-expressing cells, the latter being also increased in number in Arx KO. However, due to the co-expression of Ghrelin with several other intestinal hormones (GLP-1, Somatostatin), it is unclear whether Ghrelin-expressing cells do correspond to a distinct enteroendocrine subtype. Interestingly, Ghrelin^+^ cell numbers also increase in Nkx2.2- or *Pax4*-deficient embryonic intestines [14], [21], two genes that have been suggested to directly regulate ghrelin expression [21], [25]. However, in the current study the increase in the number of Ghrelin^+^ cells does not result from a down-regulation of Nkx2.2 or Pax4, since mRNA levels of both genes do not change in the small intestine and colon of newborn *Arx*-deficient mice. Since a co-detection of Glucagon and Ghrelin is frequent in immature developing alpha-cells in the embryonic pancreas [26], another possibility could be that Ghrelin^+^ cells might correspond to L-cells precursors (GLP1-negative) blocked in their differentiation.

Arx and Pax4 have been demonstrated to have antagonistic functions during the specification of islet sub-types in the pancreas. We therefore postulated that similar mechanisms could operate in the intestine. We found that in the intestine, the different enteroendocrine cell types similarly and differentially require Arx and Pax4. Indeed, *Gip*, *Cck*, *Sct*, *Gast*, and *Nts* intestinal expression are reduced in both knockout mouse, suggesting that the differentiation of the corresponding enteroendocrine subtypes relies on both Pax4 and Arx. On the other hand, Serotonin-expressing EC- and Somatostatin-expressing D-cell development requires Pax4 (but not Arx), while GLP1-expressing L-cells and Cholecystokinin-expressing CCK-cell differentiation depend on Arx (but not Pax4). In contrast to our results, Larsson and colleagues reported a decreased number of duodenal CCK-cells in the absence of Pax4 [15]. This difference may result from the use in the latter study of an antibody recognizing both secretin and gastrin peptides. Most importantly, we report for the first time a significant increase of *Glp1* expression, in the small intestine and colon of *Pax4*-deficient newborns. In agreement with these observations, morphometric analysis revealed an increase of GLP-1 L-cells in the ileum. The simultaneous loss of GLP1^+^ L-cells and augmented number of Somatostatin^+^ D-cells in Arx mutant mice and the opposite phenotype noted in Pax4 mutants suggest that Arx/Pax4 play antagonistic roles in enteroendocrine progenitor to promote D- or L-cell fates respectively as reported in the pancreas for the alpha- versus beta-/delta-cell destinies. Furthermore, these results support a model where D- and L-cells would differentiate from a common D/L precursor upon promotion by Pax4 or Arx, respectively. Interestingly and in contrast to *Arx*-deficient mice, Chromogranin A mRNA is reduced in Pax4 KO. This result suggests that the increase in GLP1^+^ or Ghrelin^+^ cell numbers is not sufficient to compensate for the loss of other enteroendocrine cell types. Alternatively, Pax4 could have a function in cell fate specification as well as a more general pan-endocrine role and regulate generic programs conferring endocrine properties such as the implementation of the secretory machinery. The latter hypothesis is supported by the fact that based on our lineage tracing experiments all enteroendocrine cells seem to derive from Pax4-positive progenitors.

In the embryonic pancreas, Arx and Pax4 instruct endocrine progenitors towards either an alpha- or beta-/delta-cell fate through a mutual inhibition between Pax4 and Arx [20]. Although we showed here that Arx and Pax4 have also antagonistic functions in enteroendocrine subtype specification in the intestine, this inhibitory cross-regulatory mechanism does not seem to operate exactly like in the pancreas. Indeed, while Arx is significantly upregulated in *Pax4*-deficient intestine, which could contribute to the excess of L-cells, the overall Pax4 expression is not affected in Arx mutants. However, because Pax4 is expressed early in all enteroendocrine cell precursors, such as in Serotonin^+^ precursors, which are abundant and do not express Arx, it is possible that we could not detect an increased Pax4 expression that would occur only in a minor subpopulation. Surprisingly, the expression of most of the other transcription factors, known to control enteroendocrine cell differentiation, does not change significantly in Arx or Pax4 mutants apart from *Foxa1* and *Foxa2*, which are up-regulated in *Arx*-deficient small intestine. Recently, Foxa1 and Foxa2 were identified as positive regulators of L- and D-cell differentiation [13]. Both transcription factors activate the *glucagon* promoter, stimulating the transcription of the preproglucagon mRNA encoding Glucagon, GLP-1 and GLP-2 [27], [28]. Therefore, one hypothesis could be that Arx represses *Foxa1* and *Foxa2* in endocrine progenitors, both genes being subsequently activated in L-cells to control terminal differentiation. Thus, the increased expression of *Foxa1/Foxa2* in *Arx*-deficient mice could contribute to the increased number of D-cells, since both genes have been postulated to control Isl-1 which transactivates the *Sst* promoter [29]. In the pancreas, the ectopic expression of Arx or Pax4 is sufficient to reprogram β- to α-cell and conversely [18], [19]. Surprisingly gain of function experiments of Arx and Pax4 in the intestinal endocrine cell line STC-1 did not result into any phenotypic alteration. These results suggest that Arx and Pax4 are necessary but not sufficient to promote enteroendocrine subtype features or alternatively they reflect the limitation of this experimental system.

In conclusion, our study reveals that Arx and Pax4 are similarly and differentially required for enteroendocrine cell differentiation downstream of the proendocrine transcription factor Neurog3 by controlling subtype specification and number. Our results also provide evidence that Arx and Pax4 antagonistically regulate L- and D-cell fate specification, respectively. Arx represses Foxa1/Foxa2 while Pax4 represses Arx. In humans, Aristaless-related homeobox gene (ARX) mutation leads to several neurological disorders. Other reported symptoms can include severe growth retardation, abnormal genitalia, disregulation of glycemia and intractable diarrhea [24], [30], [31], [32]. We propose that impaired enteroendocrine cell differentiation may be the cause of the chronic diarrhea in *ARX*-deficient patients as others and we reported similar phenotype in mice and patients with a mutation in Neurog3 and lacking enteroendocrine cells [5], [33]. Finally, considering the key role of enteroendocrine cells and hormones in nutrient sensing, food intake and glucose homeostasis, it would potentially be of interest in the future to find means to modulate the ratio of various enteroendocrine cell types in the adult mouse intestine and determine the consequences on energy metabolism. Such studies could stimulate novel therapeutic strategies to treat metabolic disorder, such as obesity and type-2 diabetes.

## Materials and Methods

### Animals

Animal experiments were supervised by G. Gradwohl (agreement N° C67-59 approved by the direction des Services Vétérinaires, Strasbourg, France) in compliance with the European legislation on care and use of laboratory animals. Pax4 and Arx null mice were described previously [16], [17]. Embryos were considered to be at E0.5 day of gestation at noon of the day the vaginal plugs were detected. Animals of both sexes were analyzed except in Fig. 3A were only females were studied. Unless otherwise indicated adult mice were analyzed at 3–6 months of age. *Arx* and *Pax4* heterozygous mice are kept on a 129/Sv background and Arx or Pax-deficient mice and littermates analyzed at P1–2. Expression studies described in Figure 1 and 2 were performed on CD1 mice.

### Immunohistochemistry and *in situ* hybridization

Tissues were fixed in 4% PFA, o/n at 4°C, washed in PBS, equilibrated in 20% sucrose at 4°C and embedded in OCT compound. The following primary antibodies were used: guinea pig anti-Neurog3 at 1∶1000 (provided by M. Sander, University of California - San Diego, La Jolla, CA, USA), chicken anti-beta-galactosidase at 1∶5000 (Abcam), rabbit anti-Arx at 1∶500, goat anti-ChgA at 1∶200 (Santa Cruz), goat anti-GLP1 at 1∶100 (Santa Cruz), rabbit anti-GLP1 at 1∶500 (Phoenix), goat anti-GIP at 1∶100 (Santa Cruz), rabbit anti-GIP at 1∶500 (Phoenix), goat anti-CCK at 1∶50 (Santa Cruz), rabbit anti-CCK/Gastrin at 1∶750 (provided by C. Roche, Inserm U865 Lyon, France), goat anti-Gastrin at 1∶50 (Santa Cruz), goat anti-Somatostatin at 1∶200 (Santa Cruz), mouse anti-Ghrl at 1∶1500 (Catherine Tomasetto, IGBMC, Strasbourg, France), rabbit anti-Ghrl at 1∶1000, rabbit anti-Serotonin at 1∶1000 (Diasorin Incstar), rabbit anti-Neurotensin at 1∶500 (Phoenix) and rabbit anti-PYY at 1∶500 (Phoenix). Secondary antibodies conjugated to DyLight-488, DyLight-549 and DyLight-649 (Jackson ImmunoResearch Laboratories) were used at 1∶500. For anti-Arx and anti-beta-galactosidase staining, signal amplification was performed using biotin anti-rabbit or anti-chicken coupled antibody at 1∶500 (Jackson ImmunoResearch Laboratories) and streptavidin-Cy3 conjugate at 1∶500 (Molecular Probes). Nuclei were stained with DAPI and slides were mounted in Aqua- Poly/Mount (Polysciences). RNA *in situ* hybridization were performed as previously described [34]. The following cRNA probes were used: *Pax4* (kindly provided by Dr P. Gruss) and *Arx* (Eurexpress, template ID T50123).

### Real time PCR analysis

Total RNA from the whole small intestine (duodenum, jejunum and ileum) and colon was extracted using TRIzol Reagent (Invitrogen). Reverse transcription was performed using Transcriptor Reverse Transcriptase (Roche). Quantitative PCRs were performed using mouse-specific TaqMan primers and probes (Applied Biosystems) recognizing Neurog3 (Mm00437606_s1), Chga (Mm00514341_m1), Arx (Mm00545903_m1), Pax4 (Mm01159036_m1), Pax6 (Mm00443081_m1), Pdx1 (Mm00435565_m1), Foxa1 (Mm00484713_m1), Foxa2 (Mm00839704_mH), Insm1 (Mm02581025_s1), Rfx6 (Mm00624115_m1), Neurod1 (Mm01280117_m1), Mafb (Mm00627481_s1), Pyy (Mm00520715_m1), Nts (Mm00481140_m1), Cck (Mm00446170_ m1), Sct (Mm00441235_g1), Gip (Mm00433601_m1), Gcg/Glp1 (Mm00801712_m1), Tph1 (Mm00493794_m1), Gast (Mm00772211_g1), Sst (Mm00436671_m1), Ghrl (Mm00445450_m1), Muc2 (Mm00458299_m1), Gfi1 (Mm00515855_m1) or UPL probes #20 (Roche) for Nkx2.2 (5′ primer gcagcgacaacccctaca, 3′ primer atttggagctcgagtcttgg) with TaqMan Light Cycler 480 Probes Master Mix (Roche) on Light Cycler 480 (Roche). Gene expression levels were normalized to β-actin (4352933E).

### Morphometric analysis

Somatostatin+ cells and Ghrelin+ cells were counted after immunostaining on approximately 60 sections of the duodenum at P1 on 3 Arx^+/+^ and 3 Arx^−^ samples. For GLP1+ cells, approximately 30 sections of the ileum at P1 were counted, on 3 Pax4^+/+^ and 3 Pax4^−^ samples. The numbers of hormone+ cells were normalized according to the area of the sections estimated by the surface of DAPI staining.

### Gain of function studies in STC-1 cells

STC-1 cells were transfected with 2 µg of pCAG-Arx-IRES-b-gal, pCAG-Pax4-IRES-b-gal and pCAG-GFP as control. 48 h after transfection *Arx*, *Pax4* and *hormones* mRNA levels were quantified by RT-qPCR as described above and normalized to *Rplp0* (TaqMan assay Rplp0; Mm01974474_gH).

### Statistics

Values are presented as mean ± SD. P values were determined using the 2-tailed Student's t test with unequal variance. P values of less than 0.05 were considered significant. ***, p<0.001, **, p<0.01, * p<0.05.

## Supporting Information

Figure S1 **Arx-expressing cells are located in the intestinal crypts in the adult mouse intestine.** Intestinal sections were stained with an anti-Arx antibody. Red arrows point to Arx-positive cells.
(TIF)

Figure S2 **Arx is expressed in post-mitotic cells in intestinal crypts.** Sections of adult mouse small intestine were stained with an anti-Arx antibody (revealed in red) and an anti-Ki-67 antibody (revealed in green). A representative image of an Arx-positive/Ki-67-negative nucleus found in the small intestine crypt compartment is shown.
(TIF)

Figure S3 **GLP1, GIP, CCK, Gastrin, Nts and PYY cells are lost in** ***Arx*** **-deficient mice.** Immunostaining of wild-type and P2 *Arx*-mutant mice (small intestine sections) using antibodies against intestinal peptides and serotonin. Hormone^+^ cells are green.
(TIF)

Figure S4 **Ghrl is detected in some GLP1^+^ cells and Sst^+^ cells.** Co-immunostaining of Ghrl and GLP1 or Sst on intestinal sections of wild-type adult mice. Yellow arrows point to co-expressing cells.
(TIF)

Figure S5 **Expression of** ***Neurog3*** **,** ***Neurod1*** **,** ***Rfx6*** **,** ***Mafb*** **and** ***Nkx2.2*** **mRNAs is not affected in** ***Arx*** **- or** ***Pax4*** **-deficient small intestine.** Quantification of mRNAs encoding key endocrine transcription factors in *Arx*- and *Pax4*-deficient small intestine. Real time PCR analysis in *Arx*- (n = 5) and *Pax4*- deficient mice (n = 4) and control small intestine and colon, 2 days after birth.
(TIF)

Figure S6 **Arx and Pax4 over-expression (OE) in STC-1 enteroendocrine cell line.** STC-1 cells were transfected with plasmids expressing Pax4, Arx or GFP under the control of the CAG (Cytomegalovirus enhancer/chicken β-actin) promoter. 48 h after transfection overexpression of *Arx* and *Pax4* was measured by mRNA quantification in Arx (A) and Pax4 (B) transfected cells (upper panels). A 1500- and 400-fold increase of *Arx* or *Pax4* was observed after transfection with Arx or Pax4 –expression plasmids respectively when compared to GFP-transfected STC-1 cells. (C) The expression of mRNAs encoding enteroendocrine hormones did not show significant variation upon Arx or Pax4 OE suggesting that neither Arx nor Pax4 is able to promote endocrine differentiation or hormone gene transactivation in STC-1 cells. *Tph1* mRNA, encoding Tryptophan hydroxylase 1 the rate-limiting enzyme in Serotonin synthesis, was used to evaluate the induction of Serotonin producing cells. Values represent means of fold changes (Arx-transfected/GFP-transfected or Pax4-transfected/GFP-transfected) of 3 independent experiments ± SD.
(TIF)

Table S1 **Hormone mRNA levels in the small intestine and colon of** ***Arx*** **- and** ***Pax4*** **-deficient mice at P2.** Table summarizing RT-qPCRs data presented in figure 3 and 6. Results are compared to controls and expressed in fold change. *Tph1* mRNA, endoding Tryptophan hydroxylase 1 the rate-limiting enzyme in Serotonin synthesis, was used to evaluate Serotonin producing cells. n = 4–5 for mutants and controls, Student's T-test *p<0.05, **p<0.01, ***p<0.001.
(TIF)
