# Supplementary figures and images for: The Homeodomain-Containing Transcription Factors Arx and Pax4 Control Enteroendocrine Subtype Specification in Mice
Source: PLoS One. 2012 May 3;7(5):e36449. doi: 10.1371/journal.pone.0036449 (PMC3343025; doi:10.1371/journal.pone.0036449)

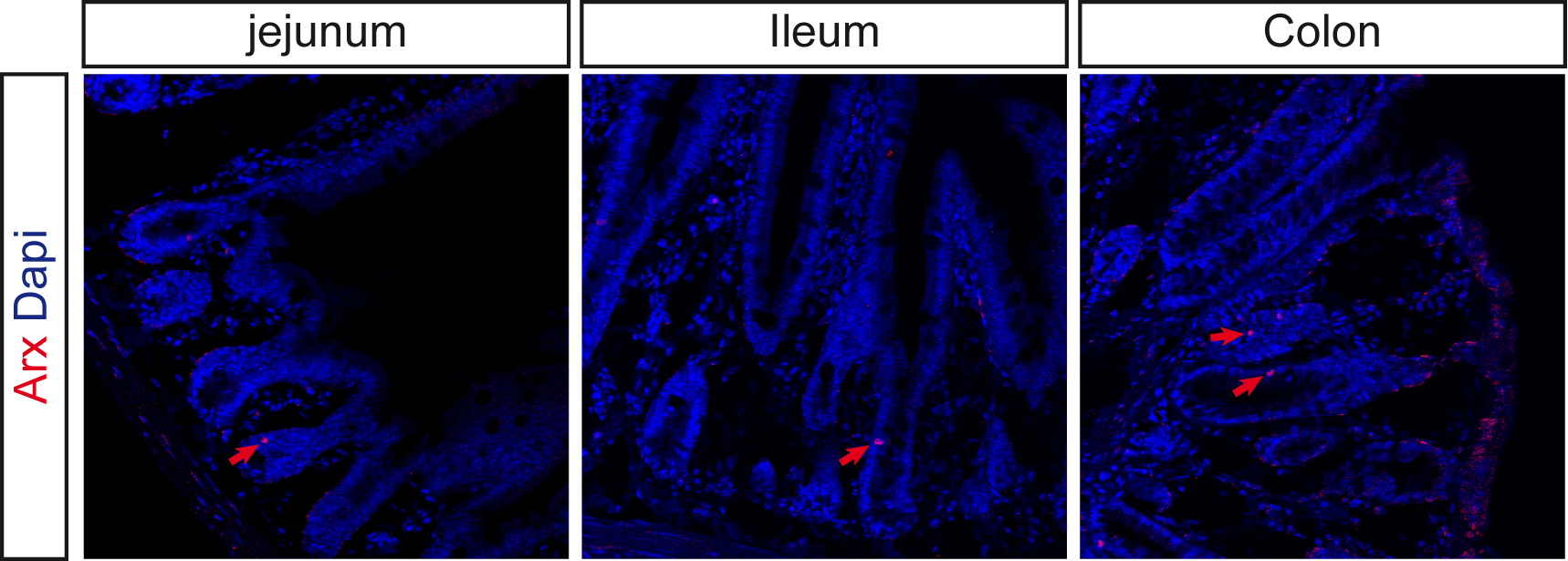

Supplement: Figure S1 — Arx-expressing cells are located in the intestinal crypts in the adult mouse intestine. Intestinal sections were stained with an anti-Arx antibody. Red arrows point to Arx-positive cells. (TIF) [file pone.0036449.s001.tif]

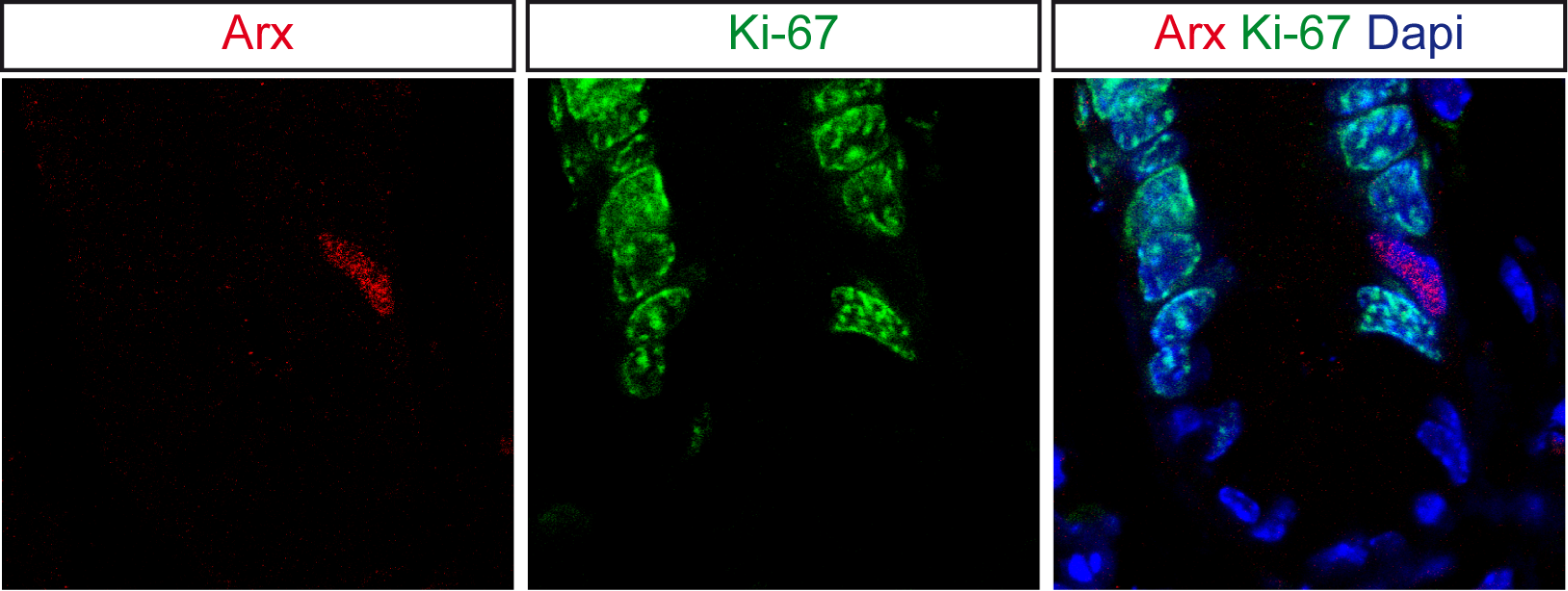

Supplement: Figure S2 — Arx is expressed in post-mitotic cells in intestinal crypts. Sections of adult mouse small intestine were stained with an anti-Arx antibody (revealed in red) and an anti-Ki-67 antibody (revealed in green). A representative image of an Arx-positive/Ki-67-negative nucleus found in the small intestine crypt compartment is shown. (TIF) [file pone.0036449.s002.tif]

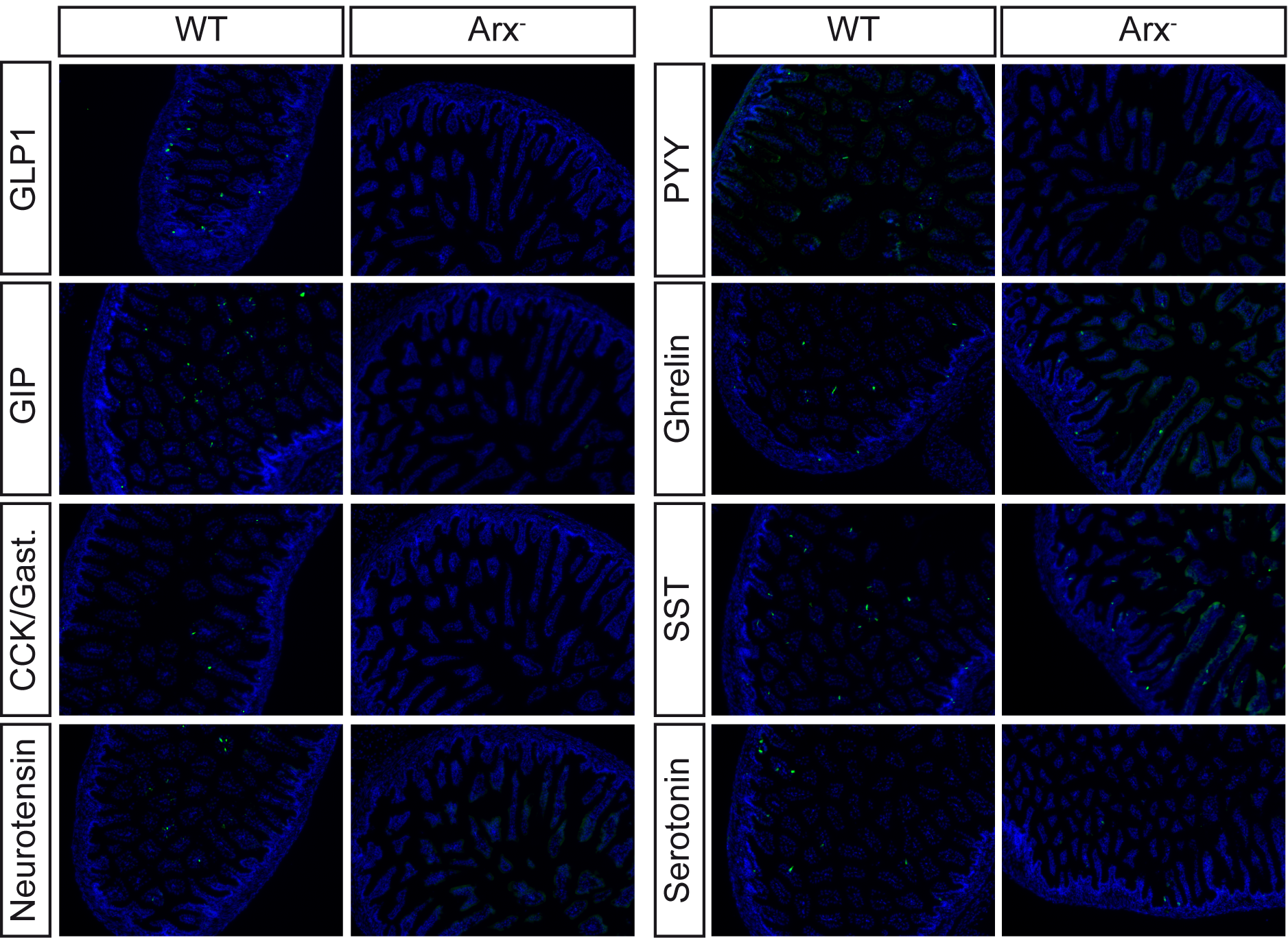

Supplement: Figure S3 — GLP1, GIP, CCK, Gastrin, Nts and PYY cells are lost in Arx -deficient mice. Immunostaining of wild-type and P2 Arx-mutant mice (small intestine sections) using antibodies against intestinal peptides and serotonin. Hormone+ cells are green. (TIF) [file pone.0036449.s003.tif]

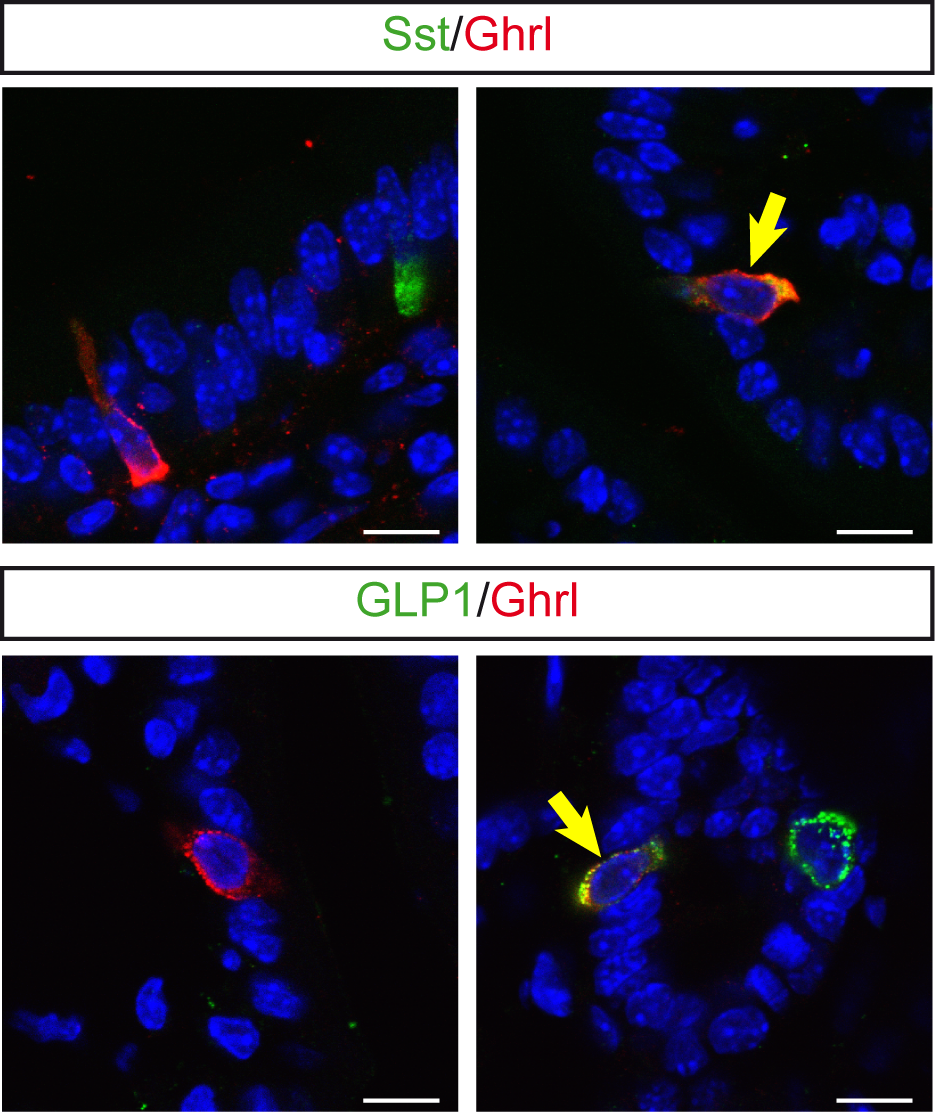

Supplement: Figure S4 — Ghrl is detected in some GLP1+ cells and Sst+ cells. Co-immunostaining of Ghrl and GLP1 or Sst on intestinal sections of wild-type adult mice. Yellow arrows point to co-expressing cells. (TIF) [file pone.0036449.s004.tif]

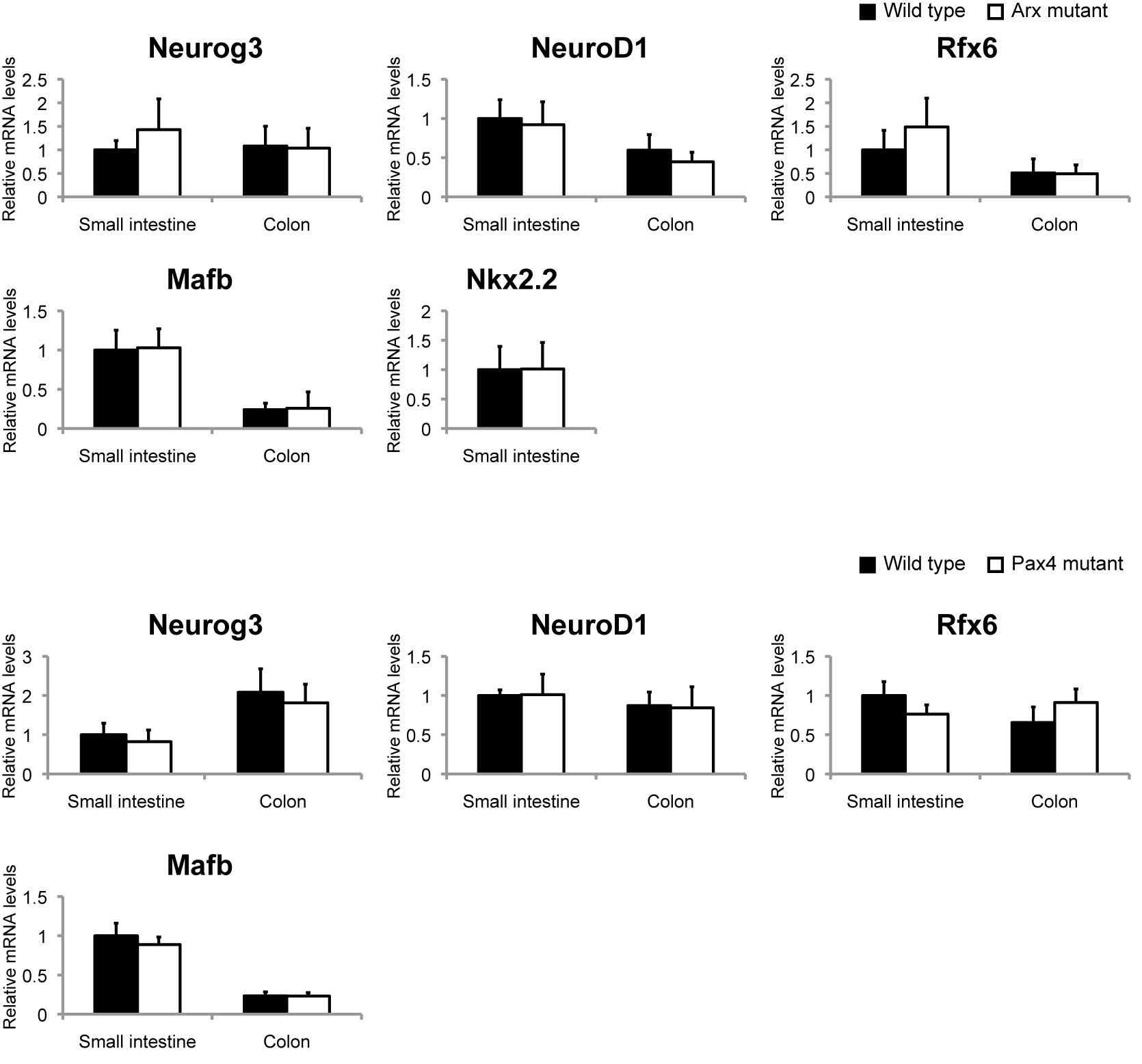

Supplement: Figure S5 — Expression of Neurog3 , Neurod1 , Rfx6 , Mafb and Nkx2.2 mRNAs is not affected in Arx - or Pax4 -deficient small intestine. Quantification of mRNAs encoding key endocrine transcription factors in Arx- and Pax4-deficient small intestine. Real time PCR analysis in Arx- (n = 5) and Pax4- deficient mice (n = 4) and control small intestine and colon, 2 days after birth. (TIF) [file pone.0036449.s005.tif]

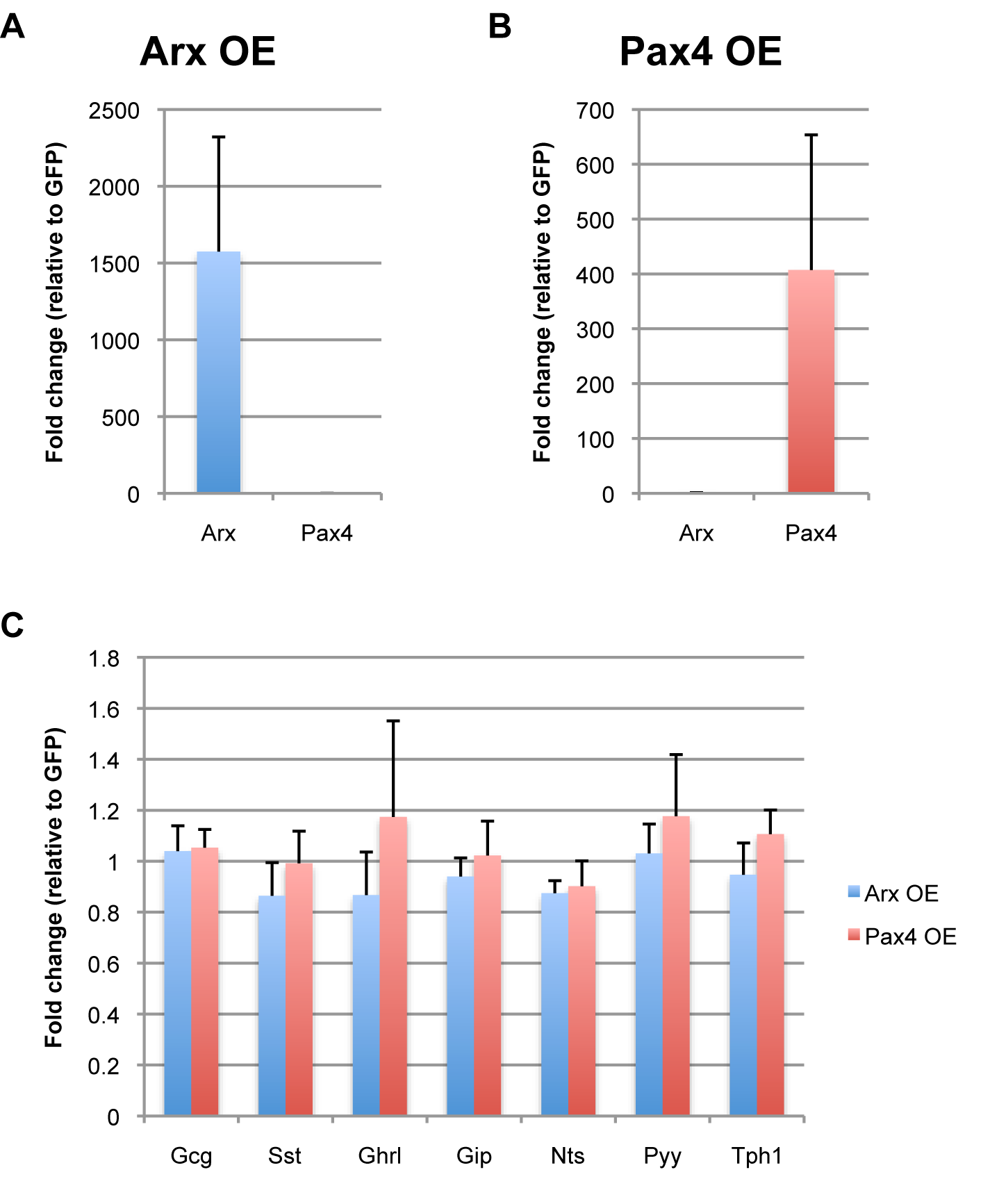

Supplement: Figure S6 — Arx and Pax4 over-expression (OE) in STC-1 enteroendocrine cell line. STC-1 cells were transfected with plasmids expressing Pax4, Arx or GFP under the control of the CAG (Cytomegalovirus enhancer/chicken β-actin) promoter. 48 h after transfection overexpression of Arx and Pax4 was measured by mRNA quantification in Arx (A) and Pax4 (B) transfected cells (upper panels). A 1500- and 400-fold increase of Arx or Pax4 was observed after transfection with Arx or Pax4 –expression plasmids respectively when compared to GFP-transfected STC-1 cells. (C) The expression of mRNAs encoding enteroendocrine hormones did not show significant variation upon Arx or Pax4 OE suggesting that neither Arx nor Pax4 is able to promote endocrine differentiation or hormone gene transactivation in STC-1 cells. Tph1 mRNA, encoding Tryptophan hydroxylase 1 the rate-limiting enzyme in Serotonin synthesis, was used to evaluate the induction of Serotonin producing cells. Values represent means of fold changes (Arx-transfected/GFP-transfected or Pax4-transfected/GFP-transfected) of 3 independent experiments ± SD. (TIF) [file pone.0036449.s006.tif]

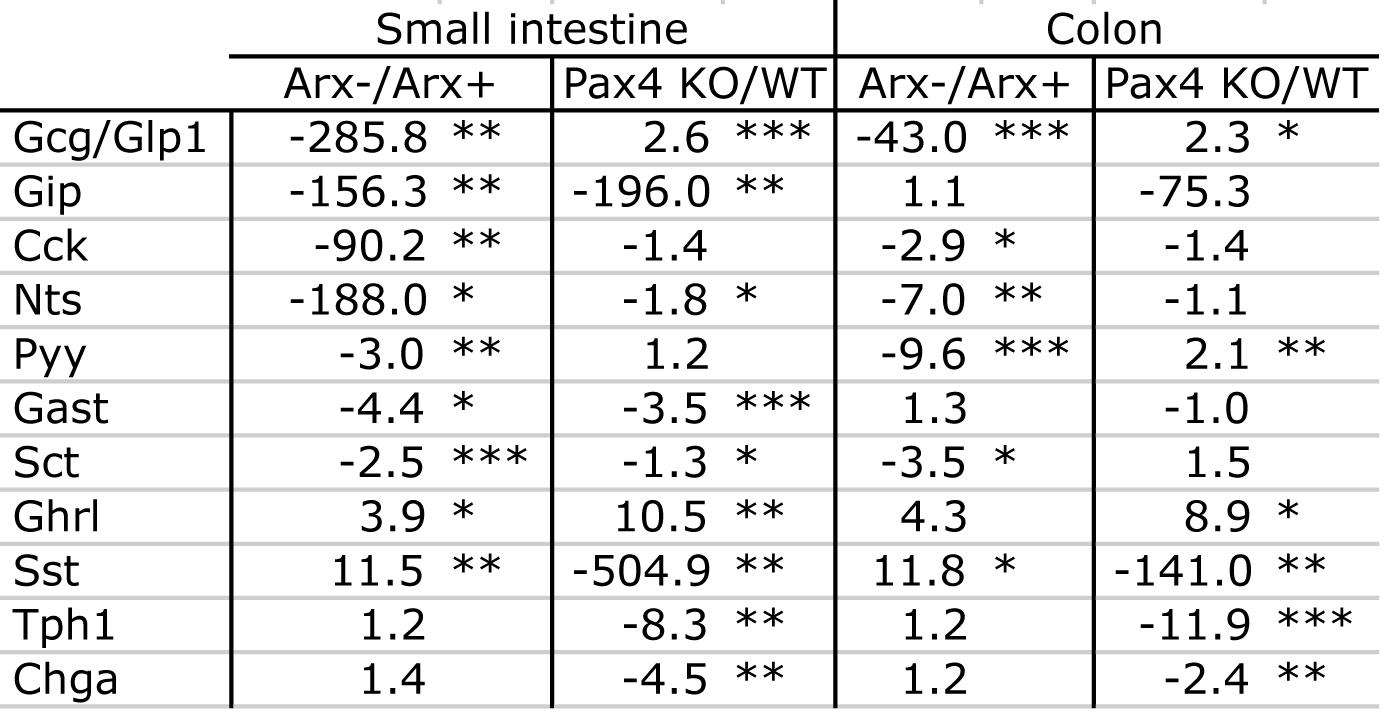

Supplement: Table S1 — Hormone mRNA levels in the small intestine and colon of Arx - and Pax4 -deficient mice at P2. Table summarizing RT-qPCRs data presented in figure 3 and 6. Results are compared to controls and expressed in fold change. Tph1 mRNA, endoding Tryptophan hydroxylase 1 the rate-limiting enzyme in Serotonin synthesis, was used to evaluate Serotonin producing cells. n = 4–5 for mutants and controls, Student's T-test *p<0.05, **p<0.01, ***p<0.001. (TIF) [file pone.0036449.s007.tif]
